# Supplementary material for: Content-rich biological network constructed by mining PubMed abstracts
Source: BMC Bioinformatics. 2004 Oct 8;5:147. doi: 10.1186/1471-2105-5-147 (PMC528731; doi:10.1186/1471-2105-5-147)
Supplement: Additional File 2 — The original results of the above study (non-essential files are deleted to keep the file size under the limit set by BMC bioinformatics). [file 1471-2105-5-147-S2.bz2 › chilibotAdditionalFile2/dip20/512ID9565586E10814/html/AR_BAG1.html]

 


 **AR** and **BAG1** 
  
Found 14 abstracts in PubMed, retrieved 14.  
 

 What does Google say? 
 PDF only 
| .edu only 

---

**Interactive relationship** (e.g. stimulation, inhibition, etc)

**Inhibitory relationship**- A C terminal region deletion mutant of BAG  [ **BAG1** ]  1L failed to co immunoprecipitate with  **AR**  and functioned as a trans dominant inhibitor of BAG  [ **BAG1** ]  1L, impairing  **AR**  induced transactivation of ARE containing reporter plasmids.  Ref: 9565586 J Biol Chem, 1998
- In addition, BAG  [ **BAG1** ]  1L significantly reduced the concentrations of 5alpha dihydrotestosterone DHT required for  **AR**  activity but did not induce ligand independent transactivation.  Ref: 9565586 J Biol Chem, 1998
**Stimulatory relationship**- BAG  [ **BAG1** ]  1L protein enhances androgen receptor  [ **AR** ]  function.  Ref: 9565586 J Biol Chem, 1998
- Although Bag1L, Bag1M, and  **Bag1**  can bind the androgen receptor  [ **AR** ]   **AR**  in vitro, only Bag1L enhanced  **AR**  transcriptional activity.  Ref: 11278763 J Biol Chem, 2001
- The effects of BAG  [ **BAG1** ]  1L on  **AR**  could not be explained by detectable alterations in the DHT induced translocation of  **AR**  from cytosol to nucleus, nor by BAG  [ **BAG1** ]  1L induced increases in the amounts of  **AR**  protein.  Ref: 9565586 J Biol Chem, 1998
**Neutral relationship**- BAG  [ **BAG1** ]  1L also markedly improved the ability of  **AR**  to transactivate reporter genes when cells were cultured with DHT in combination with the anti androgen cyproterone acetate.  Ref: 9565586 J Biol Chem, 1998
- Forced nuclear targeting of Bag1M, but not  **Bag1**  or Bag1S, resulted in potent  **AR**  coactivation, indicating that Bag1M possesses the necessary structural features provided it is expressed within the nucleus.  Ref: 11278763 J Biol Chem, 2001
- The recently identified BAG 1  [ **BAG1** ]  long BAG  [ **BAG1** ]  1L protein, an isoform of BAG 1  [ **BAG1** ]  that arises from translation initiation at a noncanonical CUG codon, was co immunoprecipitated with androgen receptors  **AR**  from LNCaP prostate cancer cells and other cell lysates.  Ref: 9565586 J Biol Chem, 1998
- RESULTS Air bag  [ **BAG1** ]  alone users had more severe overall injury severity score > or = 15 9 vs 5, chest abbreviated injury score AIS  [ **AR** ]  > or = 3 5 vs 1, and abdominal injuries AIS  [ **AR** ]  > or = 3 6 vs 0.  Ref: 9305303 South Med J, 1997

**Non-interactive relationship** (e.g. studied together, co-existance, homology, etc.)

- Given its proposed involvement in multiple cell signaling events as a regulator of Raf1, Bcl2, or androgen receptor  [ **AR** ] , we wondered whether  **Bag1**  functions in vivo as a negative regulator of Hsp70.  Ref: 10629065 Mol Cell Biol, 2000
- BAG  [ **BAG1** ]  1L, but not BAG 1  [ **BAG1** ]  or BAG  [ **BAG1** ]  1M RAP46, also markedly enhanced the ability of  **AR**  to transactivate reporter gene plasmids containing an androgen response element ARE in PC3 prostate cancer and other cell lines.  Ref: 9565586 J Biol Chem, 1998
- Based on repeated isolations, high field infection rates and abundance, Culex appeared to be the vectors of BAG  [ **BAG1** ] , BBK,  **Ar**  D 65239 BAG  [ **BAG1** ]  like, and WN viruses, Ae.  Ref: 11476327 J Med Entomol, 2001
- These findings implicate BAG  [ **BAG1** ]  1L in the regulation of  **AR**  function and may have relevance to mechanisms of prostate cancer resistance to hormone ablative and anti androgen therapy.  Ref: 9565586 J Biol Chem, 1998
- ochraceus of RVF virus, Mansonia of WN and BAG  [ **BAG1** ]  viruses, Mimomyia of WN and BAG  [ **BAG1** ]  viruses, and Phlebotomine of SAB, CHP,  **Ar**  D 95537, and GF viruses.  Ref: 11476327 J Med Entomol, 2001
- six Flaviviruses, Bagaza BAG  [ **BAG1** ] ,  **Ar**  D 65239, Wesselsbron WSL, West Nile WN, Koutango KOU, Saboya SAB.  Ref: 11476327 J Med Entomol, 2001
